# 

**Published:** 1871-08

**Authors:** 


**CONTENTS:**

ORIGINAL COMMUNICATIONS:

ARTICLE I. Intraocular Ossification. By F. B. Norcom, M.D ............ 449

ART. II. Report of Three Cases of Vesico-Vaginal Fistula. By A. Reeves Jackson, M.D ..... ....... 454

ART. III. Excisions of Head of Os Humeri for Gun Shot Wounds. By Chas. M. Clark, M.D. (Continued) ........................ 462

ART. IV. Clinical Lectures on Diseases of the Ear. By E. L. Holmes, M.D. (Continued) ....... ........ 465

ART. V. Fracture of the Cranium. By Theo. W. Stull, M.D ......... 469

ART. VI. "Formula, No. 2." By H. W. Richardson, M.D .......... 471

SELECTIONS:

The Mineral Springs of Michigan... 473

Opium ............ ................ 477

Differential Diagnosis of Acute Phthisis and Typhoid Fever .... 487

Experiments of the Effects of Alcohol on the Human Body..... .... 490

Acute Scleriasis; Spontaneous Recovery. Scleroderma; Treatment by Galvanization .................. 493

On Elixirs Containing Iron......... 498

EDITORIAL ........................ .. 500

LOOT ..................................... 506

PROCEEDINGS OF SOCIETIES ............ 510

